# Commentary: Iron deficiency of pregnancy - a new approach involving intravenous iron

**DOI:** 10.1186/s12978-018-0536-1

**Published:** 2018-06-22

**Authors:** Michael Auerbach

**Affiliations:** 1 Auerbach Hematology and Oncology, Baltimore, MD USA; 2 Georgetown University, Washington DC, USA

**Keywords:** Iron deficiency, Anemia of pregnancy, Intravenous Iron, Neonatal Iron deficiency

## Abstract

Iron deficiency anemia of pregnancy is common, especially in South Asia, and is associated with adverse maternal and fetal outcomes including increased incidences of maternal mortality, preterm labor and low birth weight. Screening for anemia alone is not sufficient to diagnose iron deficiency. Iron deficiency in neonates is associated with a statistically significant increment in cognitive and behavioral abnormalities which persist after iron repletion. Oral iron is the frontline standard but is associated with an unacceptably high incidence of gastrointestinal adverse events leading to poor adherence. Prospective evidence reports an incidence of neonatal iron deficiency up to 45% even with oral iron supplementation. New evidence reports oral iron ingestion increases serum hepcidin leading to decreased absorption suggesting further decreasing efficacy. Published evidence reports that intravenous iron is safe and effective in the second and third trimesters of pregnancy. Intravenous iron is the preferred route when there is oral iron intolerance or in those situations where oral iron is ineffective or harmful. Intravenous iron is also preferred if the anemia is severe (< 8 g/dL) in the second trimester or at any time in the third trimester when there is little expectation that adequate quantities of iron will be delivered to the fetus as iron requirements increase in each trimester. Guidelines for maternal and neonatal screening and treatment lack consistency and differ between the United States and Europe. New formulations of intravenous iron with complex carbohydrate cores that bind elemental iron more tightly mitigating the release of large quantities of labile free iron allow the administration of complete replacement doses in 15–60 min. The preponderance of published evidence suggests that intravenous iron is underutilized in pregnancy and guidelines suggesting there is insufficient evidence to recommend the routine screening and treatment of iron deficiency in gravidas should be revisited. The major recommendation from this commentary is that in low-income countries, a trial or demonstration project to test the efficacy, safety, cost and feasibility of the administration of intravenous iron to anemic and/or iron-deficient women be undertaken.

## Background

The estimated incidence of anemia of pregnancy varies between eight and 20 %, largely dependent on the economic status of the measured populations [1–4] with some areas of India and Pakistan reporting up to 90%. In some of these areas, hemoglobin levels of 8 mg/dl are found in nearly 10% of the pregnant population despite the availability and use of oral iron and vitamin preparations (unpublished data). While often considered of minor clinical significance, iron deficiency is associated with significant adverse maternal and fetal outcomes during pregnancy with a reported two-fold increased incidence of preterm labor and a three-fold increase in the incidence of low birth weight [5]. Screening for anemia alone is not sufficient to diagnose iron deficiency. If iron deficiency and heavy vaginal bleeding are present at the beginning of pregnancy, the incidence of preterm labor is increased five-fold [2]. Iron deficient mothers, irrespective of anemia, are at risk of delivering iron deficient neonates [6–8]. Recent data suggest that iron deficient neonates exhibit delayed growth and development as well as a statistically significant increment of cognitive and behavioral abnormalities which persist even after iron repletion [9]. Oral iron therapy, the current frontline standard, is often not optimal for iron deficiency in pregnancy. Intravenous iron is safe, effective and should be considered early in the treatment paradigm for iron deficient gravidas, irrespective of the presence or absence of anemia.

The Centers for Disease Control (CDC), American College of Obstetricians and Gynecologists (ACOG) and the United States Preventive Service Task Force (USPSTF) all recommend routine screening for anemia during pregnancy and the CDC and ACOG recommend low-dose iron supplementation for all pregnant women [5, 10, 11]. In all of these guidelines there remains no admonition to screen for iron deficiency at the beginning of pregnancy or at any time thereafter in the absence of anemia. These recommendations were supported by a recent USPSTF publication which reported that “there is insufficient evidence that routine prenatal screening and supplementation for iron deficiency anemia improves maternal or infant clinical health outcomes, but supplementation may improve maternal hematologic indices” [12]. While these recommendations are syntactically accurate due to the lack of published outcome data, a plethora of published evidence suggests the recommendations be revisited. This position is supported by The Cochrane Collaboration, reporting that despite the high incidence and burden of disease associated with iron deficiency anemia of pregnancy, there “is a paucity of quality trials assessing clinical maternal and neonatal effects of iron administration in pregnant women with anemia [4]. That being said, while prospective studies may be absent for proving that routine screening and supplementation is beneficial, ample data exist to recommend revisiting the USPSTF conclusions. In 1967 Scott and Pritchard reported that 58% of healthy 18 year old, non-pregnant, women have absent hemosiderin on marrow aspiration, suggesting low iron stores [13]. The current USPSTF recommendations will subsequently miss all those with iron deficiency without anemia. Pregnant women presenting to their obstetricians are not screened for iron deficiency unless anemic with a low hemoglobin concentration. Clinicians may miss up to 55% of iron deficient gravidas with significantly abnormal values when iron parameters are not added to the screening laboratory tests [14]. Iron deficiency in pregnancy precedes anemia. Further, multiple studies report significant morbidity from iron deficiency, which may occur in the absence of anemia. Examples of sequelae in the fetus include neonatal and childhood brain growth and developmental abnormalities with adverse effects on myelination, neurotransmitters and brain programming [15]. Additionally, a two-fold increased incidence of preterm birth, a three-fold increase in low birth weight, and small for gestational age infants have been reported [5]. Published evidence indicates that low serum maternal ferritin concentrations are associated with iron deficiency in neonates [16] and associated with a statistically significant increment in both cognitive and behavioral abnormalities which appears to be long lasting and detectable up to 19 years of age [9, 17]. These conclusions are supported by evidence that in 6–24 month old infants with iron deficiency anemia there is increased risk for poorer cognitive, motor, social-emotional and neurophysiologic development [18]. In addition to negative effects on the fetus, maternal iron deficiency anemia is associated with increased risk for caesarean section, increases in transfusion, perinatal bleeding, pre-eclampsia, placental abruption, abnormal maternal thyroid status, impaired wound healing, cardiac failure and death [19–21].

## Iron repletion

**Fig. 1:**
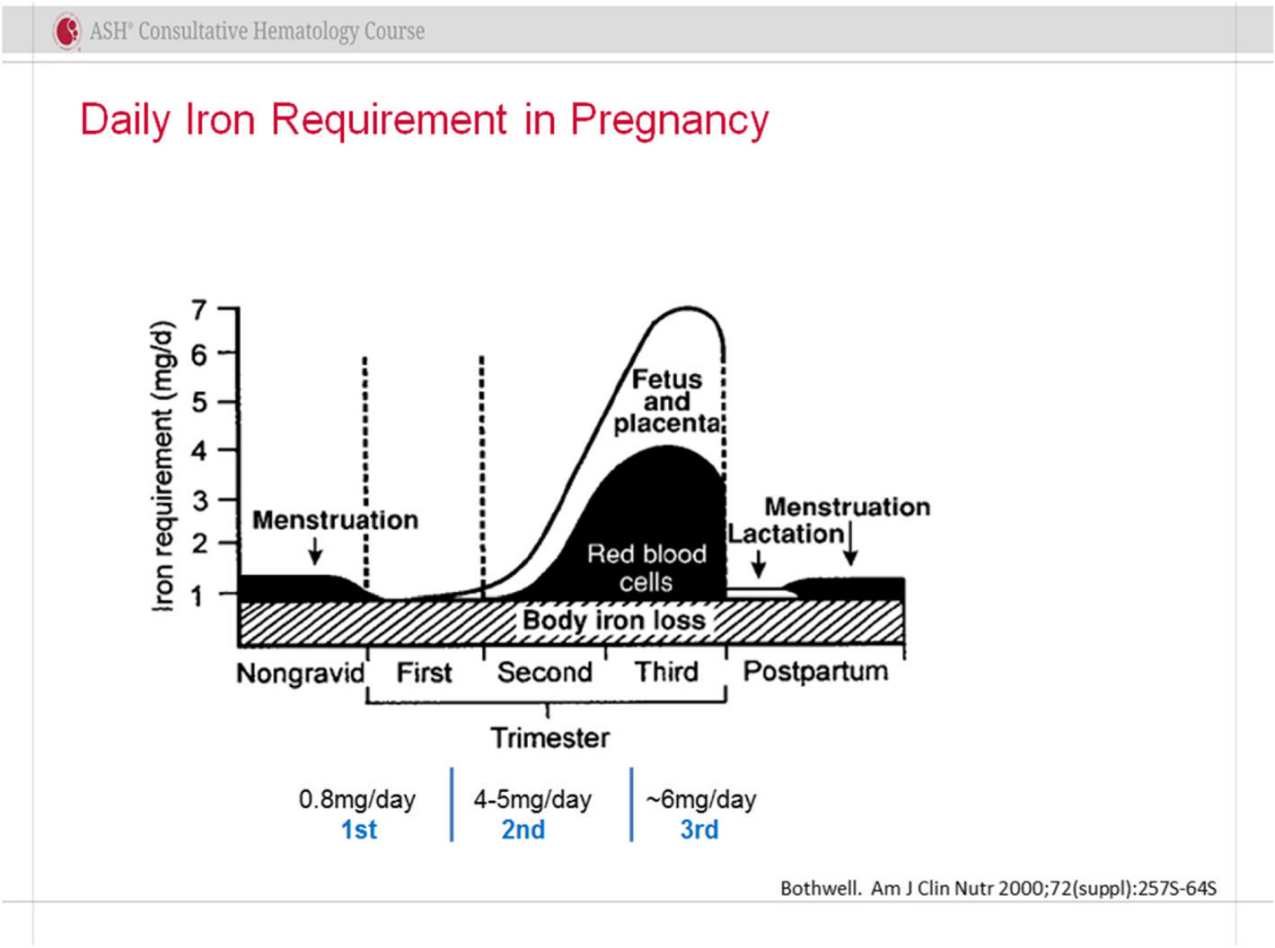
Iron requirements throughout pregnancy

Iron repletion has been shown to decrease morbidity in those with heavy uterine bleeding, inflammatory bowel disease, chronic kidney disease, cancer and chemotherapy induced anemia, heart failure, hereditary hemorrhagic telangiectasia, bariatric surgery, the pre-, peri- and post-operative periods and in critically ill patients. It is therefore reasonable to infer that in the absence of harm, a similar benefit to iron deficient gravidas would be observed leading to the credible conclusion that, given the absence of quality prospective outcomes data, we should err on the side of repletion until such data are available. This conclusion is additionally supported by a study of 2400 urban Chinese women which reported that up to 45% of infants were iron deficient despite oral supplementation [6]. Iron requirements dramatically increase in pregnancy to accommodate an expanding red cell volume, growing fetus and placenta plus any anticipated or unanticipated blood loss at delivery. This is of special significance if a caesarian section is required. Published evidence states that daily iron requirements increase from 0.8, 4–5 to 6 mg/day in the first second and third trimesters respectively [7] (Fig. 1). It is estimated that Iron requirements in pregnancy may exceed 1000 mg, with 500 mg required for red cell expansion, 300–350 mg for the developing fetus and placenta, with variable losses at delivery [8].

The World Health Organization estimates that the worldwide incidence of anemia of pregnancy approaches 50% [19]. It defines anemia of pregnancy as a hemoglobin level of less than 11 g/dL, or hematocrit < 33%, at any time during pregnancy with the CDC defining anemia of pregnancy as Hb < 11 g/dL, or hematocrit < 33% during the first and third trimesters and less than 10.5 g/dL, or a hematocrit < 32% in the second trimester [5, 22].

The current standard for iron deficiency is oral iron administered as two to three 325 mg tablets containing approximately 50–65 mg elemental iron daily. Gastric acid is necessary to conjugate the iron to amino acids, sugars and vitamin C which protects the elemental iron from conversion to ferric hydroxide in the proximal duodenum. This would be unabsorbable as a result of the massive alkaline secretions of the pancreas necessary for normal absorption. Parenthetically, this information mitigates use in gravidas who have undergone bariatric surgery with either roux-en-Y or biliopancreatic bypass procedures. A variety of other formulations such as heme polypeptide, enteric coated and timed release iron, ostensibly designed to increase tolerability, have been compared to ferrous sulfate with no improvement in toxicity and with equivalent efficacy.

**Fig. 2:**
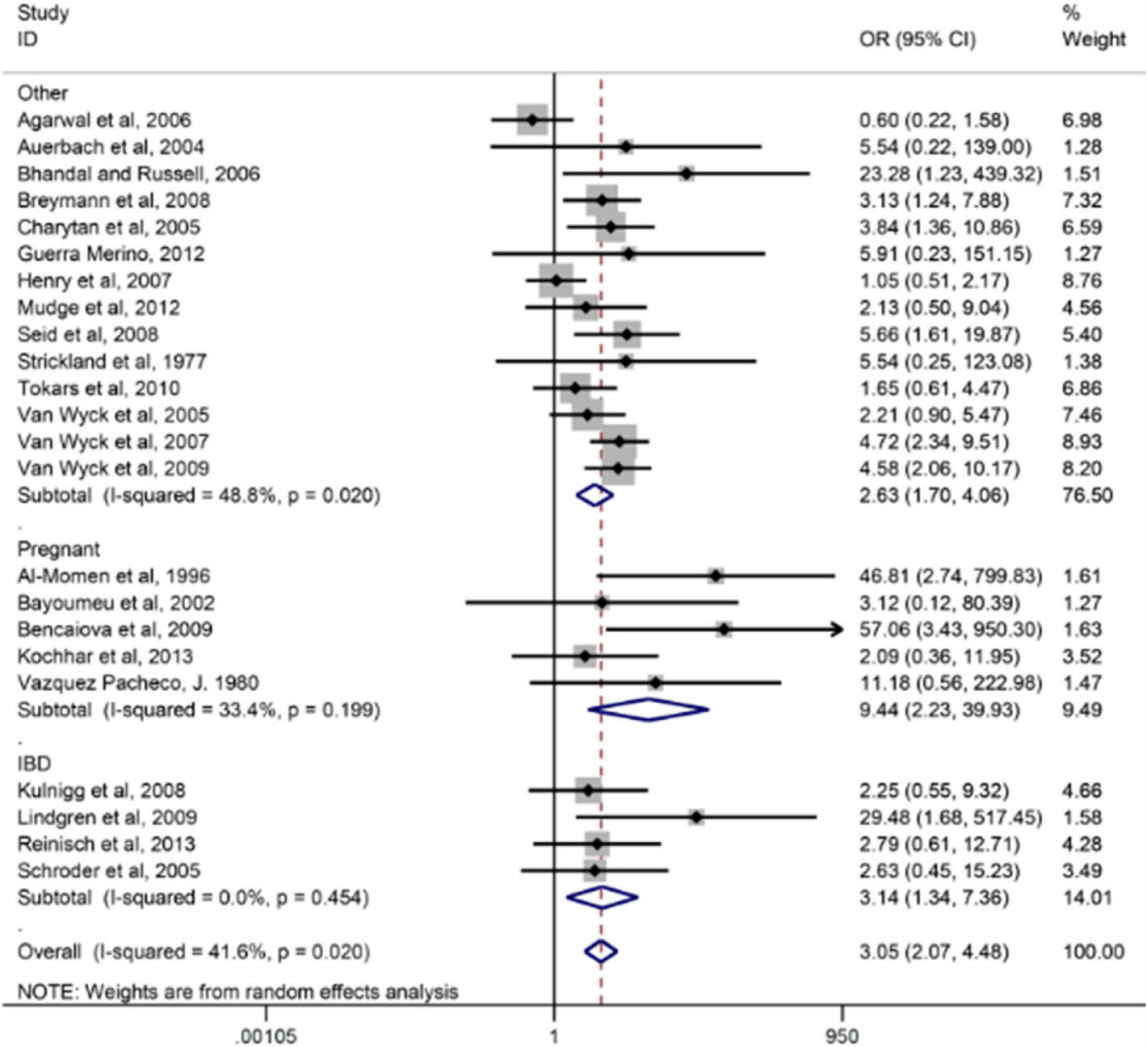
Effect of daily ferrous sulfate supplementation on the incidence of gastrointestinal side-effects in intravenous iron-controlled randomized control trials. With Permission: Tolkien et al. [23]

Oral iron is inexpensive, readily available, and easy to obtain. However, more than 70% of those to whom it is prescribed, complain of significant gastrointestinal perturbation which includes metallic taste, gastric irritation, and worsening constipation resulting in poor adherence [23] (Fig. 2). These symptoms are especially onerous as constipation is rife in pregnancy due to the rapidly increasing progesterone levels which slow bowel transit, and the enlarging uterus pressing posteriorly on the rectum. Further complicating the use of oral iron is recently published evidence demonstrating increments in serum hepcidin levels for approximately 48 h after ingestion of an iron tablet (which impair iron absorption and release) [24]. Hepcidin, the hepatic synthesized iron regulatory protein, decreases iron absorption at the level of the intestinal epithelium and release from iron laden circulating macrophages. Recently published supporting evidence using radiolabeled oral ferrous sulfate reported improved absorption with a single tablet on alternate days compared to use of daily or twice daily ferrous sulfate [25]. While this gradual repletion may suffice in an individual who is not pregnant, the need in pregnancy is more urgent. It is not credible to expect rapid, clinically meaningful iron repletion, essential for the developing fetus, with a small dose of oral iron.

Lending further support to the inadequacy of oral iron in many gravid women is published evidence reporting that a ferritin level less than 15 ng/ml in the mother compromises iron status of the growing fetus whose iron requirements for normal brain development are maximal from week 34 on [6]. Current guidelines do not recommend routine screening for iron deficiency in newborns. Yet, current guidelines also do not recommend routine prenatal screening and supplementation for iron deficiency in the absence of anemia [12]. These issues of concern question the current paradigm of frontline oral iron therapy in moderately to severely anemic pregnant women after the first trimester and suggest that the parenteral route may be a preferred option.

The use of intravenous iron in pregnancy remains sporadic at best. Whereas intravenous iron has been shown to be nearly uniformly safe and effective, with serious adverse events extremely rare, numerous published trials, irrespective of which of the currently available intravenous iron formulations is used, recommendations for its use vary greatly. The 2008 ACOG Practice Bulletin recommends intravenous iron in the “rare patient who cannot tolerate or will not take modest doses of oral iron” with the caveat that patients with severe malabsorption may benefit from parenteral iron [11]. In contradistinction, the 2012 United Kingdom guidelines states that “parenteral iron should be considered from the second trimester onwards and during the postpartum period for women with confirmed iron deficiency who fail to respond to or are intolerant of oral iron” [26]. And, in a recent review of the treatment of anemia in pregnancy, Achebe and Gafter-Gvili recommend intravenous iron for oral iron intolerant 2nd and 3rd trimester patients, 2nd trimester gravidas with hemoglobin concentrations less than 10.5 g/dL and in all in the third trimester with iron deficiency anemia. India, has no country-wide recommendations related to intravenous iron use, although some of the states, including Karnataka, where this conference took place, recommend its use in certain circumstances. There are no existing guidelines for the treatment of non-anemic, iron deficient pregnant women [22].

The successful use of intravenous iron in pregnancy is hardly new. In 1964 and in 1973, two different studies of intravenous iron in iron deficient gravidas reported the safety and efficacy in more than 2500 pregnant women, of complete replacement dosing with iron dextran in a single setting [27, 28]. Nearly ubiquitous efficacy was observed without any serious adverse events. Yet over 50 years later, the first United States prospective study of intravenous iron in pregnancy was recently published [21]. Seventy-four oral iron intolerant, second and third trimester iron deficient gravidas were questioned for oral iron intolerance, and if present, were treated with intravenous iron. All received 1000 mg of low-molecular weight iron dextran in 250 ml normal saline over 1 h without premedication, unless multiple drug allergies or asthma was present. In this case, methylprednisolone was administered prior to the test dose. Fifteen minutes after a test dose, the remainder was infused over the balance of 1 h and no serious adverse events were observed. Those enrolled were called at one, two and 7 days to assess for delayed reactions. Four weeks post-infusion or postpartum, hemoglobin concentrations and iron parameters were measured. Fifty-eight of 73 women were questioned about interval growth and development of their babies. The mean pre- and post-hemoglobin concentrations were 9.7 and 10.8 g/dL (*P* < 0.00001) and ferritin of 14.5 and 126.3 ng/mL (*P* < 0.000001) respectively. While six patients experienced transient minor infusion reactions, all of which resolved without therapy, there were no serious adverse events observed. Data for 58 infants, ages 3 months to 3 years, were available. One had delayed development reported which resolved by 11 months, the remaining 57 were reported as normal. None were diagnosed with iron deficiency. The authors concluded that intravenous iron has less toxicity and, consistent with the preponderance of published evidence, is more effective than oral iron, supporting moving its use closer to frontline therapy.

These data support a previously published observational study by the same group of 189 consecutive, non-selected, oral iron intolerant second and third trimester gravidas who received 1000 mg of low molecular weight iron dextran in 1 h [29]. Hemoglobin concentrations increased by 1–1.9 g/dL in 58% and by greater than 2 g in 24%. Anemia resolved in 95%. No serious adverse events were observed. The authors concluded that a large, single, rapidly administered dose of intravenous low-molecular weight iron dextran was effective, safe and convenient. A recent prospective international, open-label randomized controlled study compared ferric carboxymaltose to oral iron. More study participants achieved correction with the intravenous formulation without the frequent gastrointestinal side effects seen in the oral iron arm [30]. In yet another prospective comparison of low-molecular weight iron dextran and ferric carboxymaltose, the authors reported a hemoglobin rise of 2.34–2.57 g/dL at 4 weeks without any serious adverse events in either group [31] and concluded that both formulations are effective and safe, with low risk of adverse events.

It is not the purview of this review to provide an exhaustive compendium of the litany of published evidence on intravenous iron in pregnancy. However, the illustrative examples provided are consistent with the overwhelming preponderance of published data supportive of the safety and efficacy of all intravenous iron formulations in correcting iron deficiency in gravidas. A consistent finding in virtually all published evidence is the absence of serious adverse events. Why then is there such resistance to incorporate the early use of intravenous iron into the treatment paradigm of iron deficiency in pregnancy among obstetricians and gynecologists? The answer may be that the folklore of fear of serious adverse events, which include anaphylaxis, coupled with the fact that no intravenous iron formulation has been assigned the highest safety rating from FDA discourages obstetricians already struggling in a litigious environment (particularly in the United States). Misinterpretation of minor infusion reactions as a serious hypersensitivity further mitigates its use [32]. Older formulations of intravenous iron consisting of high molecular weight iron dextran, which are no longer available, were associated with an incidence of severe hypersensitivity of 1–3% [33]. While iron sucrose and ferric gluconate are safe and effective, their smaller carbohydrate cores bind elemental iron less tightly and preclude administration of doses larger than 200–300 mg in a single setting due to the release of increased amounts of labile free iron [34]. Newer formulations in the United States, Europe and Asia, which include low molecular weight iron dextran, ferric carboxymaltose, ferumoxytol, and iron isomaltoside are able to be administered as a complete replacement dose in a short single visit of 15–60 min as a result of complex carbohydrate cores which bind the elemental iron more tightly, limiting the amount of labile free iron. These formulations are associated with a lower incidence of infusion reactions than either iron sucrose or ferric gluconate and have a much lower incidence of serious adverse events, with an estimated incidence of such serious events of less than 1:250,000 doses [35].

These composite conclusions parallel results from a large meta-analysis of 103 trials comprising over 10,391 patients who were treated with intravenous iron compared to 4044 given oral iron, 1329 no iron, 3335 placebo and 155 treated with intramuscular iron [36]. In this large population, 935 women were pregnant and another 748 peripartum. Parenthetically, intramuscular iron is painful, requires multiple injections, stains the buttock and has been associated with gluteal sarcomas and subsequently should be avoided. Overall, while uncommon, infusion reactions were observed with intravenous iron although there was no increase in serious adverse events with intravenous iron compared to controls, including placebo (95% CI 0.93–1.17, 97 trial I^2^ = 9%). No difference in safety or efficacy was reported among any of the studied formulations, consistent with all prospective, intra-institutional retrospective studies and meta-analyses [37].

## Conclusions

While initial cost of intravenous iron formulations for under resourced countries may be problematic, clinical trials to determine cost effectiveness, and public pricing availability may mitigate these concerns. Therefore, the information in this commentary recommends a revisiting of the current international guidelines for the screening and treatment of anemia in pregnancy. Thus it is suggested:

1. All newly diagnosed gravidas, irrespective of hemoglobin level at presentation to their obstetricians, midwives or other providers, be screened for iron deficiency to include serum iron, total iron binding capacity, percent transferrin saturation and serum ferritin.
2. If iron deficiency is present in the first trimester one ferrous sulfate tablet every other day should be taken. We acknowledge these recommendations may not be practical for much of the world’s pregnant woman with limited health care budgets, however the potential improved outcomes may prove cost effective.
3. If iron deficiency is diagnosed in the second trimester, the hemoglobin is greater than 8 g/dL and the mother’s serum ferritin is greater than 15 ng/ml, one ferrous sulfate tablet every other day should be taken with a rapid switch to intravenous iron if the therapy proves ineffective or is poorly tolerated. If the hemoglobin level is less than 8 g/dL or the mother’s serum ferritin is less than 15 ng/ml, the intravenous route is preferable.
4. Intravenous iron is the preferred route of replacement if required, in the third trimester.
5. Neonates at risk for iron deficiency should be screened at birth. These include preterm infants, infants of diabetic mothers, infants born to anemic or iron deficient mothers, those with parasitic infestation or malaria, HIV, and those who had chronic hypoxia in utero (infants of smokers).
6. In low income countries, a trial or demonstration project to test efficacy, safety, cost and feasibility of the routine administration of intravenous iron appears prudent.
7. A prospective comparison of oral to intravenous iron with screening of neonates appears prudent.
